# The volatilome signatures of *Plasmodium falciparum* parasites during the intraerythrocytic development cycle in vitro under exposure to artemisinin drug

**DOI:** 10.1038/s41598-023-46416-1

**Published:** 2023-11-17

**Authors:** Zenaida Stead, Rosamaria Capuano, Corrado Di Natale, Arnab Pain

**Affiliations:** 1 Bioscience Program, Biological and Environmental Sciences and Engineering (BESE) Division, KAUST, 239556900 Jeddah, Saudi Arabia; 2 Department of Electronic Engineering, University of Rome Tor Vergata, Via del Politecnico 1, 00133 Roma, Italy; 3 Interdepartmental Centre for Volatilomics “A. D’Amico”, University of Rome Tor Vergata, Via del Politecnico 1, 00133 Roma, Italy

**Keywords:** Metabolomics, Microbiology

## Abstract

Volatile organic compounds (VOCs) comprise a diverse range of metabolites with high vapour pressure and low boiling points. Although they have received attention, they are a largely unexplored part of the metabolome. Previous studies have shown that malaria infections produce characteristic, definitive, and detectable volatile signatures. Many transcriptional and metabolic differences are observed at different stages of the parasite Intraerythrocytic Developmental Cycle (IDC) as well as when artemisinin-resistant parasites are put under drug pressure. This prompted our research to characterize whether these responses are reflected at a volatile level in malaria during the IDC stages using gas chromatography-mass spectrometry. We investigated whether the resistant *P. falciparum* parasites would produce their own characteristic volatilome profile compared to near-isogenic wild-type parasite in vitro*;* firstly at three different stages of the IDC and secondly in the presence or absence of artemisinin drug treatment. Finally, we explored the VOC profiles from two media environments (Human serum and Albumax) of recently lab-adapted field parasite isolates, from Southeast Asia and West/East Africa, compared to long-term lab-adapted parasites. Recognizable differences were observed between IDC stages, with schizonts having the largest difference between wild type and resistant parasites, and with cyclohexanol and 2,5,5-trimethylheptane only present for resistant schizonts. Artemisinin treatment had little effect on the resistant parasite VOC profile, whilst for the wild type parasites compounds ethylbenzene and nonanal were greatly affected. Lastly, differing culturing conditions had an observable impact on parasite VOC profile and clustering patterns of parasites were specific to geographic origin. The results presented here provide the foundation for future studies on VOC based characterization of *P. falciparum* strains differing in abilities to tolerate artemisinin.

*Plasmodium falciparum* is a protozoan parasite responsible for the most severe cases of malaria worldwide^1^. Malaria is a devastating disease affecting 247 million people worldwide and causing over 600,000 deaths in 2021^2^. Over the years *P. falciparum* has quickly developed resistance against many antimalarials produced to treat malaria, however, recent widespread concern has arisen due to emerging drug resistance to frontline artemisinin drug treatments in Southeast Asia, and more recently in Africa^3^. Outward clinical symptoms of malaria range from asymptomatic to severe whilst on a biochemical level, close interactions between the parasite and host are known to affect the host metabolic landscape^4,5^. These metabolic perturbations encompass changes in amino acids, lipids and other chemical compounds that may produce signature metabolic profiles that have the potential to act as biomarkers for malaria.

*P. falciparum* is transmitted through the bite of Anopheline mosquitoes and host odors play a key role for mosquitos to locate a human. Chemoattractant studies between *Anopheles spp* and human host have shown certain chemicals, including ammonia, lactic acid and carboxylic acids, are of significant importance for enhanced vector recruitment^6^. Pathogens have been found to manipulate host volatiles which are attuned to the olfactory cues recognized by vector species^7^. Thus, to aid malaria transmission, *Plasmodium falciparum* is thought to manipulate host odours in order to attract mosquito vectors with a profile distinct from one of general pathology^8^. Being able to identify unique plasmodium infection volatile profiles or signatures is postulated to aid in the design of novel non-invasive diagnostic tools to predict malaria infection status.

Volatile organic compounds (VOCs) are defined as a group of low molecular weight carbon-based molecules with low boiling points and high vapor pressure. VOCs are able to evaporate at ambient temperatures and pertain to a large set of chemical groups including; alcohols, ketones, aldehydes, hydrocarbons, terpenes, amines, isocyanates and sulfides^7^. Volatile samples from human subjects are often collected from sources including skin secretions, breath, urine, blood, milk, feces and saliva, with each part of the body emitting different compositions of VOCs^9^.

There is increasing evidence that particular VOC signatures can be detected depending on the disease^7^. Changes to normal cellular and organ functions due to disease or pathogens will alter host metabolic pathways and be reflected in saliva, breath and skin secretions. If unique metabolic perturbations are detected, these may be classified as specific disease biomarkers depending on the illness^10^. In vitro studies on varying pathogenic species have shown that each infected cell culture media produced its own unique characteristic VOC profiles^7,11^. Therefore, not only can VOCs be explored as a novel diagnostic method of distinguishing between different diseases, its non-invasive methodology is an advantage in diagnostic applications in healthcare.

Malaria parasites are able to influence host odour from either metabolites directly produced by the parasite and parasite-RBC interaction or indirectly with the parasite metabolites interacting with skin microbes. It has been shown that Anopheline mosquitoes are lowly attracted to freshly produced human sweat, however once incubated with skin bacteria the attraction is much higher^7,12^. VOCs released from malaria patients have been studied extensively as possible mosquito attractants, whilst VOCs released by the parasites themselves have been studied more for the direction of developing diagnostic biomarkers in malaria healthcare^7^. Reports of volatile organic compounds produced by malaria parasites have been conflicting. Kelly et al. 2015 identified 4 parasite-specific compounds, two of which were terpenes which were known compounds to be recognized by the mosquito vector *Anopheles gambiae*^6^. However, Wong et al. 2012^13^ were unable to find a difference between malaria-infected and uninfected cultures. Capuano et al. 2019 carried out VOC analysis on asexual and sexual stage parasites, results showed that sexual stages released the highest concentration of hexanal compared to all samples and concluded that different stages between sexual parasites could be distinguished based on hexanal concentration^14^. In vivo studies measuring VOCs using sorbent tubes from children in Malawi and from children in Kenya both found unique volatile signatures correlating to malaria infection^15,16^.

Artemisinin resistance is characterized by slow parasite clearance following artemisinin monotherapy or artemisinin combination therapy (ACT) where primarily the sensitivity of ring stage parasites to artemisinin is affected. In 2009 the first emerging cases of artemisinin resistance in *P. falciparum* to ACT were identified and reported around the Greater Mekong Subregion (GMS) in the Palin province, Southeast Asia^17^. In 2014, the *Plasmodium kelch13* (*PfK13*) gene was reported as a marker of resistance to artemisinin^18^. Initially twenty *PfK13* mutations were observed that were associated with the resistance phenotype, but by 2013 the *PfK13* single nucleotide polymorphism, C580Y, was the most predominant mutation being recorded in Western Cambodia^19^. In 2017, an artemisinin-resistant strain of *P. falciparum* with mutation M579I in the *PfK13* gene was reported from Equatorial Guinea and in 2021 the WHO reported evidence of *PfK13* mutations R561H (markers of resistance to artemisinin) and C469Y/A675V observed in Rwanda and Uganda respectively^20^.

Therefore, the aim of this study was to analyze volatile profiles of different *Plasmodium falciparum* isolates, both long-term lab-adapted strains with defined genotypes and recently lab-adapted field isolates from a wider geographic origin, using GC–MS to discern if there are unique signatures depending on parasite genotypes known to confer tolerance to Artemisinin drugs and geographic origin of the isolates. Volatile chemical compositions of wild type and genetically resistant *Plasmodium* parasites between ring, trophozoite and schizont stages of the parasite were compared. The volatile profiles were also measured following artemisinin drug treatment for the parasite isolates. Finally, the VOCs were determined for two separate culturing media environments, these being human serum and Albumax.

## Results

### Experimental design

Three distinct experiments were conducted to explore changes in VOC profiles produced by asexual stages of *P. falciparum* cultures under differing growth conditions. The first experiment consisted of synchronizing asexual stage parasites for two cycles prior to conducting the experiment, after which newly invaded ring stage parasites were captured and fresh media added. Supernatants were then collected at timepoints 26hpi (ring), 38hpi (trophozoite) and 48hpi (schizont). The second experiment involved supernatant collection from early ring stage cultures (10hpi) after parasites had either been treated or not treated with antimalarial drug dihydroartemisinin. The final experiment consisted of collecting supernatant after one full 48 h cycle for lab strain parasites and lab-adapted field isolates in both Albumax media and Human serum conditions. For each experiment the GC–MS data was analyzed and focused on specific objectives related to the differences between species and media. The overall experimental setup is show in supplementary Fig. S1.

Overall GC–MS identified 62 compounds across all experiments (see Supplementary Table S2 online). Among these compounds, styrene and hexanal showed the highest abundance, while most compounds were only present in trace amounts in our assay. Some of the compounds were only found in specific replicates. Therefore, in the following analysis, we focused our attention on the compounds that were present in all replicates of at least one sample.

#### Volatilome profiles of rings, schizonts, and trophozites stages

**Figure 1:**
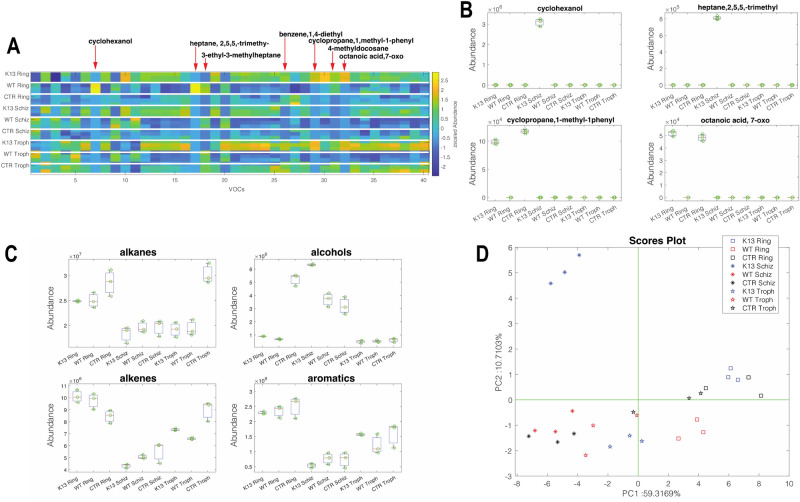
Volatilome profile of synchronized asexual wild-type (WT, NF54) and artemisinin resistant (K13, NF54-C580Y) parasite stages. (**A**) Heatmap of normalized VOCs abundance including wild-type and artemisinin resistant ring, trophozoite and schizont stages as well as media control (CTR) for each parasite stage. (**B**) Distribution of stage-specific compounds in all samples and all stage-specific controls. (**C**) Distribution of the cumulative abundance of chemical families. (**D**) PCA scores plot of data in Fig. 1A for wild-type and artemisinin resistant ring, trophozoite and schizont stages as well as media control (CTR) for each parasite stage.

In the first experiment, the volatile emissions from different stages of the synchronized asexual parasite cycle were studied. VOC patterns were measured in wild-type (WT) and artemisinin-resistant (K13, denoted by parasites carrying the C580Y mutation in the *kelch13* gene) rings, schizonts, and trophozoites. In total, these samples were characterized by 40 recurrent volatile compounds. This is the largest pool of metabolites compared to the other experiments described in this study. Such a large variety of VOCs indicates the increased and diversified metabolic processes occurring in these stages compared to individual parasites. Figure 1A shows the volatilome patterns of K13 and wild-type stages, along with their respective culture media, as a heatmap. A visual inspection of the heatmap reveals differences between the various stages. Supplementary Fig. S2 shows the distribution of the abundance of the 40 compounds in the different groups. Each compound is characterized by a dominant abundance in one group compared to the others. 19 out of 40 VOCs show the highest abundance in ring stages. However, in these samples, the contribution of culture media is not easily separated from the specific parasite VOCs. Nonetheless, there are specific cases that highlight the existence of metabolic processes correlated with artemisinin resistance. Some of these cases are shown in Fig. 1B. Cyclohexanol and 2,5,5-trimethylheptane are not released by the culture media and are only present in the headspace of K13 schizonts. 1-methyl-1-phenyl-cyclopropane and 7-oxo octanoic acid are components of ring culture media, but they appear to be completely metabolized by wild-type rings. A similar behavior is exhibited by 1,4-diethylbenzene, which is released by the culture media of rings and trophozoites but disappears in the corresponding stages of wild-type parasites. The analysis of the cumulative distribution of chemical families (Fig. 1C) shows that rings, regardless of drug resistance, are characterized by a high abundance of alkanes, alkenes, and aromatic compounds, while alcohols dominate among the schizonts. The multivariate comparison of volatilome profiles can be achieved by the Principal Component Analysis of the VOCs matrix. Figure 1D shows the plot of the first two principal components (explaining 66% of the total variance in the plot). The plot highlights the distinct volatilome pattern of K13 schizonts compared to the other groups. On the other hand, rings and trophozoite stages form close clusters where a smaller separation between K13 and wild-type samples can be observed.

#### Voilatilome profile of artemisinin treated and untreated wild-type parasites compared to resistant parasites

**Figure 2:**
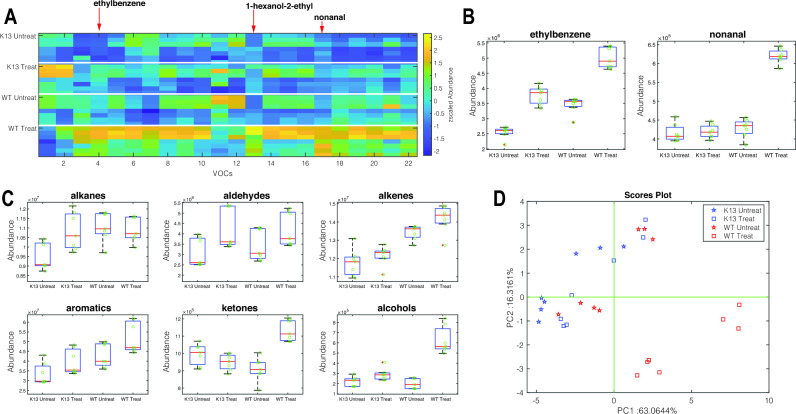
Volatile metabolites in artemisinin treated and untreated Wild-Type (NF54) and K13 artemisinin resistant (NF54-C580Y) *Plasmodium*. (**A**) the abundance of a set of recurrent 22 VOCs shown as a heatmap. To enable comparison between VOCs with different abundances, the abundance of each VOC is normalized in the [0,1] range. Each plot is headed with the smallest *p*-value returned by the Kruskal–Wallis test. (**B**) distribution of compounds showing the largest change after drug administration. (**C**) Distribution of the cumulative abundance of the major chemical families in the four groups of samples. (**D**) Plot of the first two principal components of the PCA calculated with standardized data shown in Fig. 2A.

In the second experiment, the VOC profiles of artemisinin-treated wild-type (WT) and resistant malaria strains (K13) were compared to those of untreated parasites, where the drug was administered at 6 h rings for four hours. For this analysis, the abundances of 22 VOCs found in all replicates of the same group were used. Figure 2A shows the heatmap of the intensity of the abundance of the selected VOCs. A batch effect can be observed in the heatmap due to conducting the experiment twice and having two separate runs on the GC–MS to check the results for reproducibility. The results are seen to be reproducible because similar VOCs are being attained from both runs. A quantitative variation between the runs is observed however despite the actual concentration of VOC values changing between run one and run two, the observed trend remained the same for both runs. The boxplots of the distribution of all these compounds in the four groups (WT and K13, untreated and treated) are shown in supplementary Fig. S3. In both wild-type and resistant parasites, treatment with artemisinin changes the pattern of VOCs. Wild-type parasites undergo more drastic changes. Specifically, the abundance of ethylbenzene and nonanal is greatly affected by the pharmacological treatment (see Fig. 2B). This behaviour is also evident when grouping the 22 selected VOCs according to their chemical family and then considering the total abundance of the members of each family. Figure 2C shows the distribution of cumulative VOC intensities in the four groups of samples. Artemisinin treatment increases the production of aldehydes, alkenes, aromatics, and alcohols in both parasites, while the increases in alkanes and ketones are exclusively found in artemisinin-resistant and wild-type parasites, respectively. Finally, the discussion about individual compounds can be extended to the entire pattern by calculating the principal component analysis (PCA). Figure 2D shows the scores plot of the PCA. This plot accounts for more than 80% of the total variance in the data. PCA has been calculated on standardized data (zero mean and unitary variance for each VOC) to emphasize the changes in the overall metabolic profile rather than the changes in the most abundant compounds. Treated wild-type parasites are largely separated from the other groups. This result agrees with the behavior of individual VOCs and their chemical families. In particular, levels of compounds Ethybenzene, 1-Hexanol, 2ethyl- (see Supplementary Fig. S3 online) and Nonanal are found to be significantly elevated in wild-type treated parasites compared to the rest of the conditions. In conclusion, this experiment shows that pharmacological treatment affects metabolic processes, and the magnitude of the effect is strongly dependent on the inherent resistance of the parasite to the drug.

#### Volatilome profile of long-term lab-adapted and recently lab-adapted field isolates with defined genotypes

The final experiment aimed to study the volatilome profile of the supernatant of cultures of six *P*. *falciparum* isolates, each cultured in the two different media conditions. Essential nutrients for parasite growth are usually obtained from human serum during parasite cultivation. A limited availability of human serum has resulted in the use of ‘Albumax’, a commercially produced lipid-enriched bovine albumin, as an alternative^21^. Comparing Albumax to human serum culturing conditions will show just how much parasite environment can influence the VOC profile. It was of interest to analyze the *P. falciparum* isolates from three different parts of the world and see whether the VOC profiles could be distinguished depending on the geographic origin of the parasite, as well as compare the long-term lab-adapted parasite VOC profiles to field isolate profiles. From the total pool of detected volatile compounds, the 32 compounds that were found in all the replicates of at least one group of samples were retained (see Supplementary Table S3 online).

**Figure 3:**
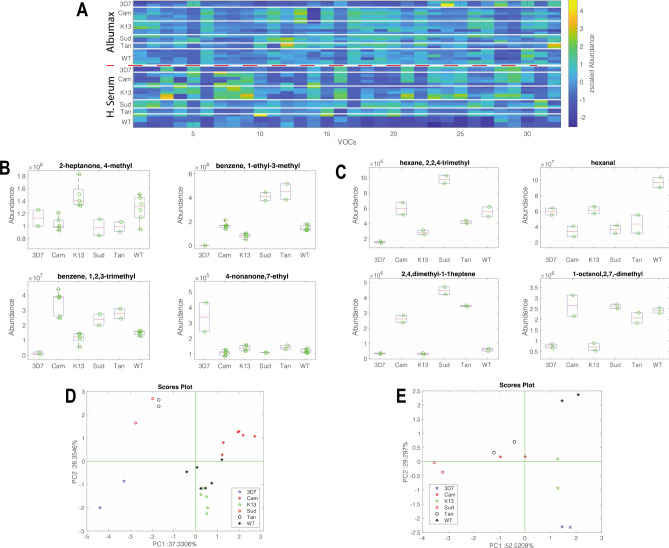
Comparison of the normalized abundance of the VOCs in the headspace of lab-adapted and field isolate parasites cultured in Albumax and Human serum. To enable comparison, the abundance of VOCs is scaled in the interval [0,1]. (**A**) Normalized abundance heatmap, the sequence of parasite is repeated in Albumax and human serum. (**B**) Distribution of the normalized abundance of the VOCs with *p* < 0.01 respect to species in Albumax. (**C**) Distribution of the normalized abundance of the VOCs with p<0.01 respect to species in Human serum. (**D** and **E**) Plots of the first two principal components calculated with the more discriminant VOCs in Albumax and human serum respectively. Lab adapted strains include 3D7, WT (NF54) and K13 (NF54 20 C580Y). Lab-adapted field isolates include Tan (MRA-1169 Tanzania), Cam (MRA-1251 Cambodia) and Sud (MRA-464 Sudan)..

Figure 3A shows the normalized abundance of the volatile compounds. Most of the compounds exhibit clear differences between the two environments. Two distinct sets of compounds are more abundant in parasites cultured in Albumax than human blood, respectively (see Supplementary Figs. S4 and S5 online). The abundance of these compounds is not directly related to the volatile organic compounds (VOCs) released by the environment itself, but rather, it is the growth in different environments that elicits different metabolite production.

A non-parametric Kruskal–Wallis rank sum test was applied to the subsets of Albumax and human serum growth parasites to determine the VOCs that discriminate between the species. Figure 3B,C show the boxplots of the compounds for which the Kruskal–Wallis test returned a *p*-value < 0.01. In both media, each species is characterized by a distinct pattern of VOCs. Only two compounds, 2,7-dimethyl-octanol and 1,2,3-trimethylbenzene, are common to both media. However, the distribution of these compounds among the species is different suggesting that the production of this metabolite depends on the environment.

A simple evaluation of the patterns of VOCs characterizing the species can be obtained by the principal component analysis (PCA) of GC–MS data. Figure 3D,E show the scores for Albumax and human serum, respectively. In both cases, the scores plot of the first two principal components shows the clustering of replicated samples of the same species. The hierarchy of clustering can be explained by the influence of the environment on the production of metabolites. The variance explained in each score plot is also different, being approximately 63% in Albumax and 81% in human serum. This difference points out a stronger correlation of the VOC profiles in human serum compared to Albumax. From this experiment, we can conclude that each *P. falciparum* isolate is characterized by a distinct VOC profile, but the quality and quantity of the volatile metabolites depend on the environment in which the parasite is cultured.

## Discussion

Volatile metabolite investigations as indicators of pathophysiological states has recently grown in popularity. The presence of *Plasmodium* parasites creates a diseased metabolic state which elicits both a quantitative and qualitative alteration of VOC profile^22^. Our experiments have shown that parasite VOC profiles can be detected reliably and reproducibly and are comparable to previous studies conducted on *Plasmodium* VOCs^7^.

As parasites grow through the asexual intraerythrocytic developmental cycle, they undergo many metabolic changes. It is well documented that throughout the IDC cycle the parasite undergoes transcriptional variations depending on the stage^23,24^. Parasites are metabolically highly active and this activity changes depending on the stage of the IDC cycle they are at^25^. Therefore, the purpose of this experiment was to see whether these changes, depending on lifecycle stage, were observed at a volatilome level and whether similar metabolites were being detected. Previous studies have proved that specific metabolites were elevated during certain *Plasmodium* stages, with hexanal found to be highly elevated in *Plasmodium* gametocytes^14^. Therefore, we investigated a synchronized asexual cycle to see if overall in the ring, trophozoite or schizont stages there was a specific metabolite or metabolic profile that distinguishes from other stages. Rings, trophozoites and schizonts had their own clustering patterns as seen from the PCA plot (Fig. 1D) regardless of whether the parasite was wild-type or artemisinin resistant. However, a large difference was seen between wild type schizonts and artemisinin resistant schizonts. Namely this difference may be caused by differences in Cyclohexanol and 2,5,5-trimethylheptane production by schizonts between the two parasite strains as these metabolites are present in significant amounts only in the artemisinin resistant parasite media. When comparing different groups of compounds in the samples; alkanes, alkenes, aldehydes, and aromatics, it was seen that these compounds were most abundant at the ring stage whilst trophozoite and schizont stages had similar abundance levels that were lower. In addition, the abundance of compounds in infected red blood cells was equivalent to levels found in the control media with red blood cells at each stage. Only for alkanes it was seen that trophozoites had a substantially lower abundance of these compounds compared to the control. Alcohols however were found at higher levels only in schizont stages which may be linked to dolichylated proteins being produced only at this later stage of the IDC^26^. Thus, looking at individual compound abundance, such as cyclohexanol, showed significant differences compared to looking at overall groups. Further work needs to be done to ascertain the importance of these chemicals especially at the schizont stage in the parasite’s metabolism.

In bulk RNA-seq and single cell RNA-seq the parasites are seen to respond to artemisinin stress, by upregulating cellular stress response pathways such as antioxidant defense and unfolded protein response^27^. We expected to see this stress perturbation reflected at a metabolite level and, this led us to investigate the volatilome profile when parasites are exposed to DHA treatment. Experiments were conducted using parasites that were both wild-type (which phenotypically show no resistance to DHA drug) and artemisinin resistant parasites (phenotypically show resistance to an artemisinin drug due to a single nucleotide polymorphism at position C580Y in the *Kelch13* gene) to see whether the metabolic response was affected depending on whether a resistant mutation was present or not. In this study we show for the first time that *Plasmodium* VOC profiles have significantly different responses to DHA treatment, despite the only difference between parasite strains is a single point mutation in the Kelch13 protein. A much larger variation was seen between the wild type treated and untreated samples, in comparison to the artemisinin resistant parasites which had much less variation in volatilome profile between samples that were either treated or not treated with artemisinin. Therefore, the resistant mutation has allowed the parasites to develop an incredibly efficient mechanism of withstanding the drug effects.

In the first two experiments, we established that the NF54 lab strain parasites can be observed to have volatilome differences in synchronized asexual stages as well as differences whether under drug pressure or not. In addition, visible differences were seen between parasites that had the sole difference of a SNP mutation in the *kelch13* gene. This led us to investigate whether recent lab-adapted field isolates from African or Southeast Asian origins also had their own volatilome profiles that could be used to distinguish them apart. In addition, the experiments were conducted in two different culturing conditions: Albumax and Human serum. Overall, compounds 2-Heptanone, 4-methyl-; 1-Octanol, 2,7-dimethyl- and Oxalic acid, bis(2-ethylhexyl) ester (see Supplementary Figs. S4 and S5 online) were seen with the most significant difference between the two culturing conditions. These VOCs were significantly elevated only in all human serum parasite samples, whilst in the controls and Albumax sample these compounds are found at lower concentrations. In both Albumax and human serum conditions there was a clear difference separating long-term lab- adapted parasites from the recently lab-adapted field isolates. In Albumax, higher levels of compounds Benzene, 1-ethyl-3-methyl-; Benzene and 1,2,3-trimethyl- (see Supplementary Fig. S4 online) were seen to be contributing to differences of Cambodia, Sudan and Tanzania parasites compared to K13, 3D7 and wild type parasites. Furthermore, there was a clear separation between the recently culture-adapted parasites with clustering seen to separate out the African field isolates away from the Southeast Asian parasites.

This effect was also replicated for the human serum grown parasites and a similar pattern was observed as seen in Fig. 3E. In human serum, higher levels of compound 2,4,-dimethyl-1,1-heptene and mesitylene were seen to be contributing to differences of Cambodia, Sudan parasites compared to K13, 3D7 and wild type parasites (Fig. 3C, Supplementary Fig. S5). Thus, these compounds may be influencing and contributing to the separation seen between samples. Therefore, these results confirm that each parasite strain displays their own unique volatilome profile, the patterns of which remain true despite a change in culturing condition and perhaps human host. The scientific impact of this may translate into an alternative method to detect parasite origin.

This research offers clues as to whether certain metabolites or metabolic trends may be translated into mosquito attractants and used as a parasite strategy for transmission. Previous studies have found that certain compounds such as hexanal and chemical group terpenes have been observed to attract *Anopheles gambiae*^6^^,^^28^. Therefore, future work would require performing experiments to understand the origin of the characteristic volatiles displayed by *P. falciparum*.

## Methodology

### Culturing of parasites

The following in vitro culture-adapted *P. falciparum* laboratory strains were used: 3D7, NF54 and NF54 C580Y for all described experiments. As of now NF54 parasites will be referred to as wild type (WT) and NF54 C580Y as resistant-K13 parasites (K13). *P. falciparum* isolates were obtained from Dr Frederic Ariey’s laboratory at Cochin institute, in Paris. Lab-adapted field isolates, obtained from BEI resources, included MRA-1169 Tanzania, MRA-1251 Cambodia, MRA-464 Sudan (See supplementary Table S1 online). Parasites were maintained in human red blood cells and cultured in RPMI 1640 complete medium containing 0.5% Albumax. For human serum experiments, parasite cultures were maintained in RPMI 1640 culture medium supplemented with 10% human serum. All cultures were synchronized with 5% D-sorbitol (w/v) treatment for 10 min at 37 °C for two or more successive growth cycles.

### Supernatant sample collection for ring, trophozoite, schizont stage VOC profile

Cultures of WT and K13 parasites were maintained in RPMI 1640 media, and cultured in flasks gassed every 48 h with 5% CO_2_ and 5% O_2_ mixture. Cultures were monitored for parasites to reach 3hpi, cultures were then synchronized with 5% D-sorbitol (w/v) treatment for 10 min at 37 °C. 20 mL of culture was used for both WT and K3, with a hematocrit of 4% and parasitemia of 8%. The experiment was performed once with one biological replicate per each stage. Post sorbitol synchronization, fresh media was added and flasks gassed for the ring stage (3hpi-26hpi). At 26hpi the supernatant was collected by centrifugation at 800 g for 10 min and immediately stored at  − 80 °C. Fresh media was re-added on top of the cells and left to continue to grow further until 38hpi, after which supernatant was again collected by centrifugation and immediately stored at  − 80 °C. This process was repeated for cells to grow until 48hpi.

### Supernatant sample collection for dihydroartemisinin (DHA) treated VOC profile

Cultures of WT and K13 parasites were maintained in either RPMI 1640 media and cultured in flasks gassed every 48 h with 5% CO_2_ and 5% O_2_ mixture. Cultures were monitored for parasites to reach 6hpi, cultures were then synchronized with 5% D-sorbitol (w/v) treatment for 10 min at 37 °C . Parasites were split into duplicate flasks containing 10 mL of culture, in 4% hematocrit and 4% parasitemia. Parasites in the untreated condition had 0.1% dimethyl sulfoxide (DMSO) added to the culture, whilst in the treated condition parasites had dihydroartemisinin (DHA 700 nM final concentration) added. Parasites were then pulse treated for 4 h after which the supernatant was collected by centrifugation at 800 g for 10 min. All supernatants were immediately transferred to -80 °C. The experiment was performed twice, each time there were two biological replicates for each condition.

### Supernatant sample collection for 48 h VOC profile- albumax and human serum

Cultures of WT, K13, 3D7, MRA-1169 (Tan), MRA-1251 (Cam), MRA-464 (Sud) parasites were maintained in either RPMI 1640 media (Albumax) or the same batch of Human serum media and cultured in flasks gassed every 48 h with 5% CO_2_ and 5% O_2_ mixture. As controls, media alone and media with 4% hematocrit were kept in a flask and gassed every 48 h. When parasites were at an early ring stage a sorbitol synchronization was performed after which fresh media was added, cells were gassed and left for 48 h in an incubator at 37 °C. 20 mL of culture was used for all samples, with a hematocrit of 4% and parasitemia of 8%. Following the 48 h incubation, supernatant was collected via centrifugation at 800 g for 10 min. All supernatants were immediately transferred to  − 80 °C. WT and K13 parasites cultured in Albumax was an experiment performed twice, each time with 2 biological replicates. 3D7, MRA-1169, MRA1251 and MRA-464 parasites cultured in Albumax was an experiment conducted once with 2 biological replicates per condition. Parasites cultured in human serum was an experiment conducted once with all parasite strains having 2 biological replicates, and MRA-464 having 1 biological sample with 2 technical replicates.

## GC–MS

*Plasmodium* culture supernatant fractions were stored at  − 20 °C until GC–MS analysis. The day before measurements, samples were defrosted at 4 °C overnight. 3 mL of Parasite culture supernatant were then transferred into a 20 ml headspace glass vial and crimped by aluminum cap with PTFE/silica septum (Chromselection, Distributed by: HTA s.r.l., Brescia, Italy).

Sample conditioning and VOC extraction by Solid Phase Micro-Extraction (SPME) were managed by the all-in-one GC autosampler HT2800T (HTA HTA s.r.l., Brescia, Italy) operating in SPME mode. Supernatants were heated at 40 °C for 1 h. During this time, a 50/30 μm Divinylbenzene/Carboxen/Polydimethylsiloxane (DVB/CAR/PDMS) fiber (SUPELCO, Bellefonte, PA, USA) was introduce in the vial headspace allowing VOCs preconcentration. The fiber was then automatically transferred at the injection port of the GC–MS where collected VOCs thermally desorbed for 3 min at 250 °C in splitless mode.

The GC–MS was the GCMS-QP2020NX model (Shimadzu, Kyoto, Japan) equipped by a capillary column SH-I-5 ms (30 m × 0.25 mm × 0.25 μm, Shimadzu, Kyoto, Japan). VOCs were separated on column, using ultra-high purity helium streamed at a constant linear velocity of 29.3 cm/s. Column temperature was programmed with the following profile: 40 °C for 5 min, increased to 220 °C at the rate of 7 °C/min, then ramped to the final temperature of 300 °C after 15 °C/min, held for 3 min. Total analysis time was 39 min. The transfer line to the MS and the ion source temperature was kept at 250 °C. Mass spectrometer is a single quadrupole operating in electron impact ionization mode, with an ionization energy of 70 eV. The detector operated with the full scan of mass ranging between 30 and 400 m/z.

GC–MS data were extracted using the section of GCMS post-run analysis of the GCMSsolutions software (version 4.0, Shimadzu Corporation), that provide to the automatic chromatogram peak integration and putative compound identification. Putative peak identification was performed using NIST20R library^29^, for mass spectrometry-based analysis. Peaks of all the chromatograms were manually aligned respect their retention time and mass spectrum base peak, comparing all the chromatograms. The aligned peak matrix, comprising the compounds obtained in all three performed experiments, contains 67 compounds. single chromatograms contain from 30 to 50 compounds.

### Supplementary Information

Supplementary Information 1.

Supplementary Information 2.

Supplementary Information 3.

Supplementary Information 4.

## Data Availability

GC–MS data are available on request from the corresponding authors.
