# Supplementary material for: The volatilome signatures of Plasmodium falciparum parasites during the intraerythrocytic development cycle in vitro under exposure to artemisinin drug
Source: Sci Rep. 2023 Nov 17;13:20167. doi: 10.1038/s41598-023-46416-1 (PMC10656521; doi:10.1038/s41598-023-46416-1)
Supplement: Supplementary file 4 — Supplementary Information 4. [file 41598_2023_46416_MOESM4_ESM.pdf]

The volatilome signatures of Plasmodium falciparum parasites during the intraerythrocytic development cycle in vitro under exposure to artemisinin drug

Zenaida Stead1, Rosamaria Capuano2,3, Corrado Di Natale2,3*, Arnab Pain1*

1. Bioscience Program, Biological and Environmental Sciences and Engineering (BESE) Division, KAUST, Jeddah, 239556900, Saudi Arabia
2. Department of Electronic Engineering, University of Rome Tor Vergata, Via del Politecnico 1, 00133 Roma, Italy.
3. Interdepartmental Centre for Volatilomics "A. D'Amico", University of Rome Tor Vergata, Via del Politecnico 1, 00133 Roma, Italy.

Supplementary Material

Parasite Strain	Country	Mutation (KELCH13)	Lab of origin
NF54	Country information not available	none	F. Ariey
NF54 C580Y	Country information not available	C580Y	F. Ariey
3D7	Country information not available	none	F. Ariey
MRA-1169	Tanzania	VBRF010000024.1:1,727,849T>G	M. Fried
MRA-1251	Cambodia	C580Y	DA. Fidock
MRA-464	Sudan	VBRF01000025.1: 1,727,988 Insertion of ATTATT	TE Wellmans

Table S1: Summary information table of parasite strains used for this study.

	Putative identification	CAS #	Similarity	Rings, schizonts, and trophozoites	Artemisinin treated wild-type and resistant parasites	Lab-adapted field isolates with different genotypes
1	2-Butanamine, (S)-	513-49-5	88%			
2	Pentanal	110-62-3	87%			
3	Octane, 3-methyl-	2216-33-3	89%			
4	Pentane, 2,3,3-trimethyl-	560-21-4	94%	X		
5	Heptane, 3-ethyl-5-methylene-	537680 (PubChem CID)	79%			
6	Heptane, 4-methyl-	589-53-7	90%	X	X	X
7	Hexane, 2,2,4-trimethyl-	16747-26-5	91%	X		X
8	Hexanal	66-25-1	93%	X	X	X
9	2,4-Dimethyl-1-heptene	19549-87-2	95%	X	X	X
10	Dimethyl sulfide	75-18-3	85%			
11	Ethylbenzene	100-41-4	89%	X	X	
12	o-Xylene	95-47-6	85%			X
13	Cyclohexanol	108-93-0	95%	X		

14	Styrene	100-42-5	97%	**X**	**X**	**X**
15	Hexanoic acid, 2-pentenyl ester, (Z)-	74298-89-8	84%	**X**		
16	2,5-Dimethylhexane-2,5-dihydroperoxide	3025-88-5	80%	**X**	**X**	**X**
17	2-Heptanone, 4-methyl-	6137-6-0	92%	**X**	**X**	**X**
18	1-Octanol, 2,7-dimethyl-	15250-22-3	85%	**X**		**X**
19	Benzaldehyde	100-52-7	91%	**X**	**X**	**X**
20	Benzene, 1,3,5-trimethyl	108-67-8	85%	**X**		**X**
21	2-tert-Butoxytetrahydrofuran	1927-59-9	86%			
22	Benzene, 1-ethyl-3-methyl-	620-14-4	91%	**X**	**X**	**X**
23	n-Caproic acid vinyl ester	3050-69-9	91%			
24	Benzene, 1,2,3-trimethyl-	526-73-8	83%	**X**	**X**	**X**
25	Heptane, 2,5,5-trimethyl-	1189-99-7	90%	**X**		**X**
26	3-Ethyl-3-methylheptane	17302-1-1	90%	**X**	**X**	**X**
27	1-Octene, 3,7-dimethyl-	4984-01-4	88%	**X**	**X**	**X**
28	1-Hexanol, 2-ethyl-	104-76-7	94%	**X**	**X**	**X**
29	Indane	496-11-7	91%	**X**	**X**	**X**
30	1-Octene, 2,6-dimethyl-	6874-29-9	75%			
31	Decane	124-18-5	75%	**X**	**X**	**X**
32	Benzene, 4-ethyl-1,2-dimethyl-	934-80-5	91%	**X**		**X**
33	Benzenemethanol, .alpha.,.alpha.-dimethyl-	617-94-7	83%	**X**	**X**	
34	Benzoic acid, methyl ester	93-58-3	92%			**X**
35	Nonanal	124-19-6	93%	**X**	**X**	
36	Benzene, 1,4-diethyl-	105-5-5	83%	**X**		
37	Benzene, 1,2,4,5-tetramethyl-	95-93-2	90%	**X**		
38	o-Cymene	527-84-4	95%	**X**		
39	4-Nonanone, 7-ethyl-	40237-88-5	79%			**X**

40	Cyclopropane, 1-methyl-1-phenyl-	2214-14-4	82%	**X**		
41	Nonane, 3,7-dimethyl-	17302-32-8	91%			**X**
42	3-Ethyl-4-methyl-3-heptanol	66719-39-9	86%	**X**		**X**
43	4-Methyldocosane	25117-30-0	82%	**X**		**X**
44	Octanoic acid, 7-oxo-	14112-98-2	82%	**X**		
45	Azulene	275-51-4	85%			**X**
46	Dodecane	112-40-3	96%	**X**	**X**	**X**
47	Sulfurous acid, decyl 2-ethylhexyl ester	6420775(PubChem CID)	90%	**X**		**X**
48	Decane, 2,3,5,8-tetramethyl-	192823-15-7	87%			
49	Oxalic acid, bis(2-ethylhexyl) ester	13675-20-2	90%	**X**		**X**
50	Benzene, 1,3-bis(1,1-dimethylethyl)-	1014-60-4	96%	**X**	**X**	
51	1-Undecene, 4-methyl-	74630-39-0	94%			
52	1-Iodo-2-methylundecane	73105-67-6	90%			
53	Nonane, 5-(2-methylpropyl)-	62185-53-9	90%			
54	Tridecane	629-50-5	96%	**X**	**X**	
55	1-Heptanol, 2-propyl-	10042-59-8	85%			
56	Octadecane, 1-(ethenyloxy)-	930-2-9	84%			
57	2,4-Decadienal, (E,E)-	25152-84-5	91%			**X**
58	2,2,6,7-Tetramethyl-10-oxatricyclo[4.3.0.1(1,7)]decan-5-one	121747-63-5	80%	**X**	**X**	
59	Cyclopentene, 5-hexyl-3,3-dimethyl-	61142-66-3	85%			
60	Not Identified	-	0%	**X**		**X**
61	Tetradecane	629-59-4	92%	**X**	**X**	**X**
62	2,5-di-tert-Butyl-1,4-benzoquinone	2460-77-7	86%			

Table S2: List of VOCs detected in at least one sample. For each VOC, the putative name and the corresponding CAS is reported. The similarity is the score in percentage of the correspondence of the detected mass spectra with NIST20R library. Crosses indicate the VOCs that was found in all replicas of at least one group of samples in the each of the three experiments.

		ALBUMAX							HUMAN SERUM						
		bkg	3D7	Cam	K13	Sud	Tan	WT	bkg	3D7	Cam	K13	Sud	Tan	WT
1	Heptane, 4-methyl-	green	green	green	green	green	green	green	green	green	green	green	green	green	green
2	Hexane, 2,2,4-trimethyl-	green	green	green	green	green	green	green	green	green	green	green	green	green	green
3	Hexanal	green	green	green	green	green	green	green	green	green	green	green	green	green	green
4	2,4-Dimethyl-1-heptene	green	green	green	green	green	green	green	green	green	green	green	green	green	green
5	o-Xylene	white	green	white	white	green	white	white	green	green	green	green	green	green	green
6	'Styrene '	green	green	green	green	green	green	green	green	green	green	green	green	green	green
7	2,5-Dimethylhexane-2,5-dihydroperoxide	green	green	green	green	green	green	green	green	green	green	green	green	green	green
8	2-Heptanone, 4-methyl-	green	green	green	green	green	green	green	green	green	green	green	green	green	green
9	1-Octanol, 2,7-dimethyl-	green	green	green	green	green	green	green	green	green	green	green	green	green	green
10	benzaldeide	green	green	green	green	green	green	green	green	green	green	green	green	green	green
11	Mesitylene	green	white	green	green	green	white	green	white	white	white	white	green	green	white
12	Benzene, 1-ethyl-3-methyl-	green	white	green	green	green	green	green	green	white	green	white	green	green	white
13	Benzene, 1,2,3-trimethyl-	green	green	green	green	green	green	green	white	green	green	green	white	green	green
14	Heptane, 2,5,5-trimethyl-	green	green	green	green	green	green	green	green	green	green	green	green	green	green
15	3-Ethyl-3-methylheptane	green	green	green	green	green	green	green	green	green	green	green	green	green	green
16	1-Octene, 3,7-dimethyl-	green	green	green	green	green	green	green	green	green	green	green	green	green	green
17	1-Hexanol, 2-ethyl-	white	white	green	green	green	green	green	white	white	white	white	white	white	white
18	Indane	green	green	green	green	green	green	green	green	green	green	green	green	green	green
19	Dodecane	green	green	green	green	green	green	green	green	green	green	green	green	green	green
20	Benzene, 4-ethyl-1,2-dimethyl-	green	green	green	green	green	green	green	green	green	green	green	green	green	green
21	Benzoic acid, methyl ester	green	green	green	green	green	green	green	green	green	green	green	green	green	green
22	4-Nonanone, 7-ethyl-	green	green	green	green	green	green	green	green	green	green	green	green	green	green
23	Nonane, 3,7-dimethyl-	green	green	green	green	green	green	green	white	green	green	green	green	green	green
24	3-Ethyl-4-methyl-3-heptanol	green	green	green	green	green	green	green	white	green	green	green	green	green	green
25	4-Methyldocosane	green	green	green	green	green	green	green	white	green	green	green	green	green	green
26	Azulene	green	green	green	green	green	green	green	green	green	green	green	green	green	green
27	Dodecane	green	green	green	green	green	green	green	white	green	green	green	green	green	green
28	Sulfurous acid, decyl 2-ethylhexyl ester	green	green	green	green	green	green	green	green	green	green	green	green	green	green
29	'Oxalic acid, bis(2-ethylhexyl) ester'	green	green	green	green	green	green	green	green	green	green	green	green	green	green
30	2,4-Decadienal, (E,E)-	green	green	green	green	green	green	green	green	green	green	green	green	green	green
31	UNIDENTIFIED	green	green	green	green	green	green	green	green	green	green	green	green	green	green
32	Tetradecane	green	green	green	green	green	green	green	green	green	green	green	green	green	green

Supplementary Table 3: Presence (green) and absence (white) of all volatile organic compounds found in Albumax cultured samples and Human serum cultured samples.

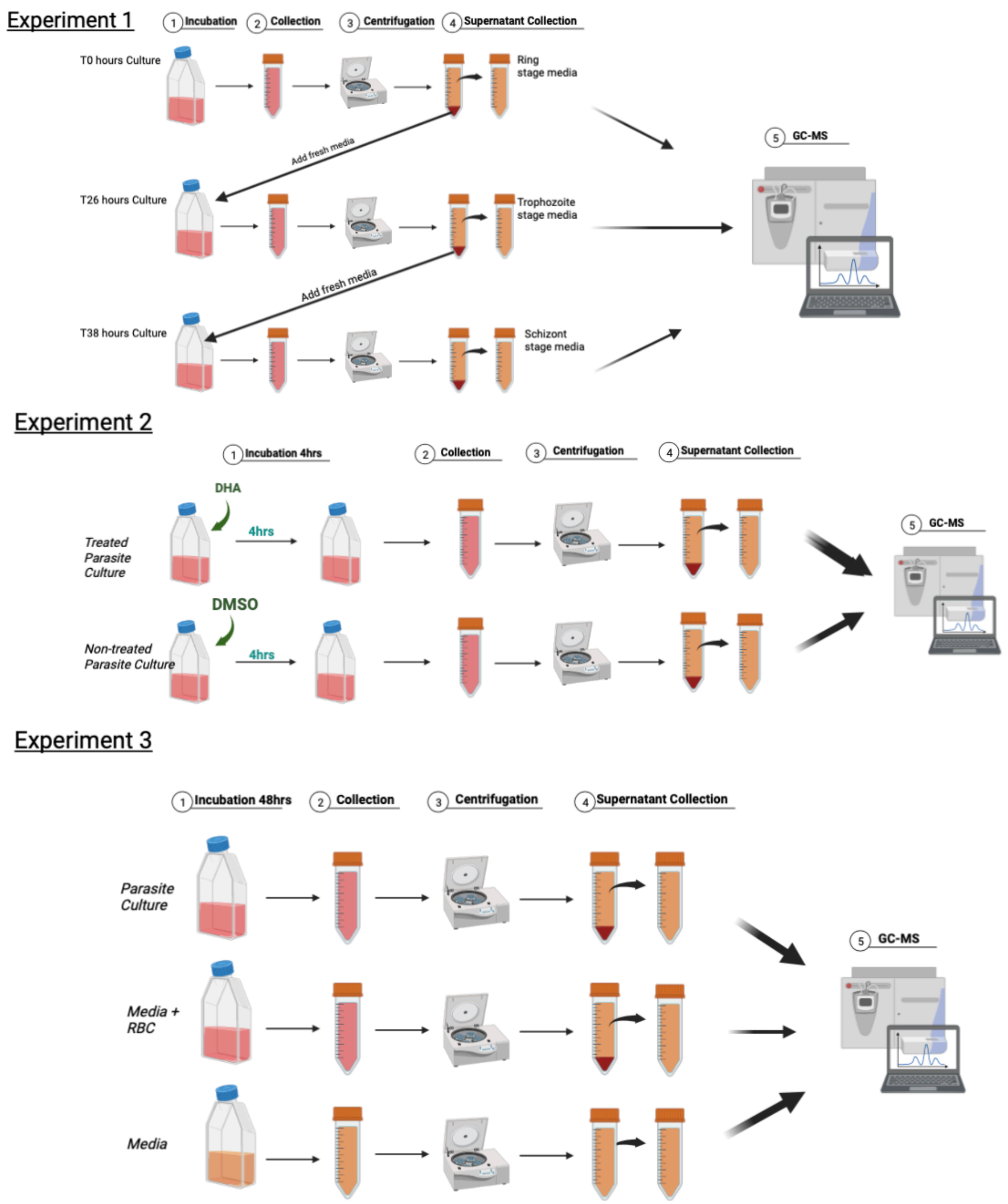

Figure S1: Experimental set up. Experiment 1: Synchronized asexual parasite cultures had fresh media added at T0 hpi. Supernatant was collected at 26hpi, fresh media was added on top of remaining iRBC culture and then collected at 38hpi. This process was repeated for final supernatant collection timepoint at 48hpi. Collected supernatants were measured using GC-MS. Experiment 2: Synchronized asexual parasite cultures at 6hpi ring stage were treated with

dihydroartemisin or DMSO for 4 hours. Supernatants were collected and measured using GC-MS. Experiment 3: Synchronized asexual parasite cultures of field isolate and lab strains were cultured for 48hrs, supernatants were collected and measured using GC-MS.

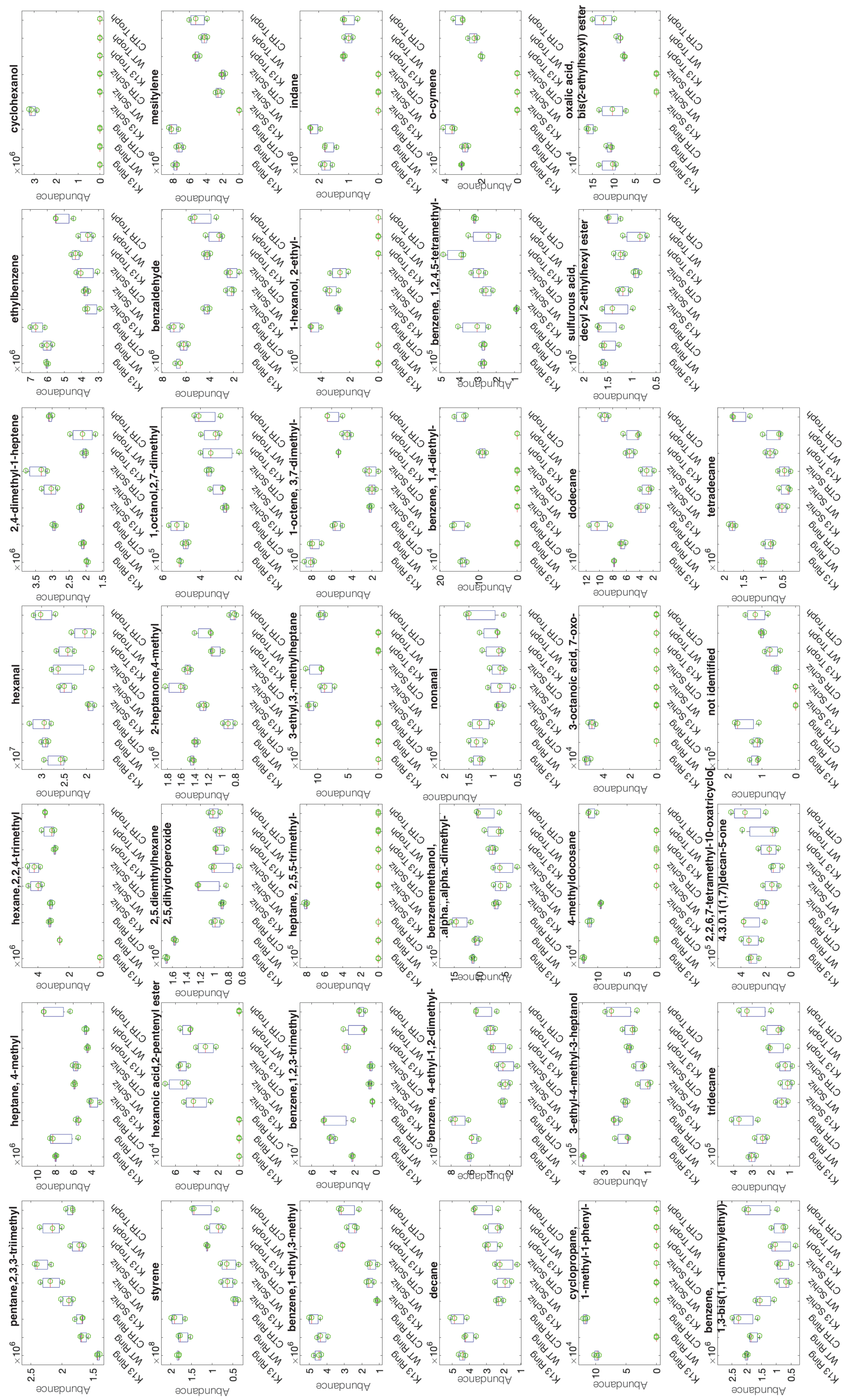

Figure S2: Distribution of the abundance of the compounds composing the Volatilomic profiles of rings, schizonts, and trophozites stages.

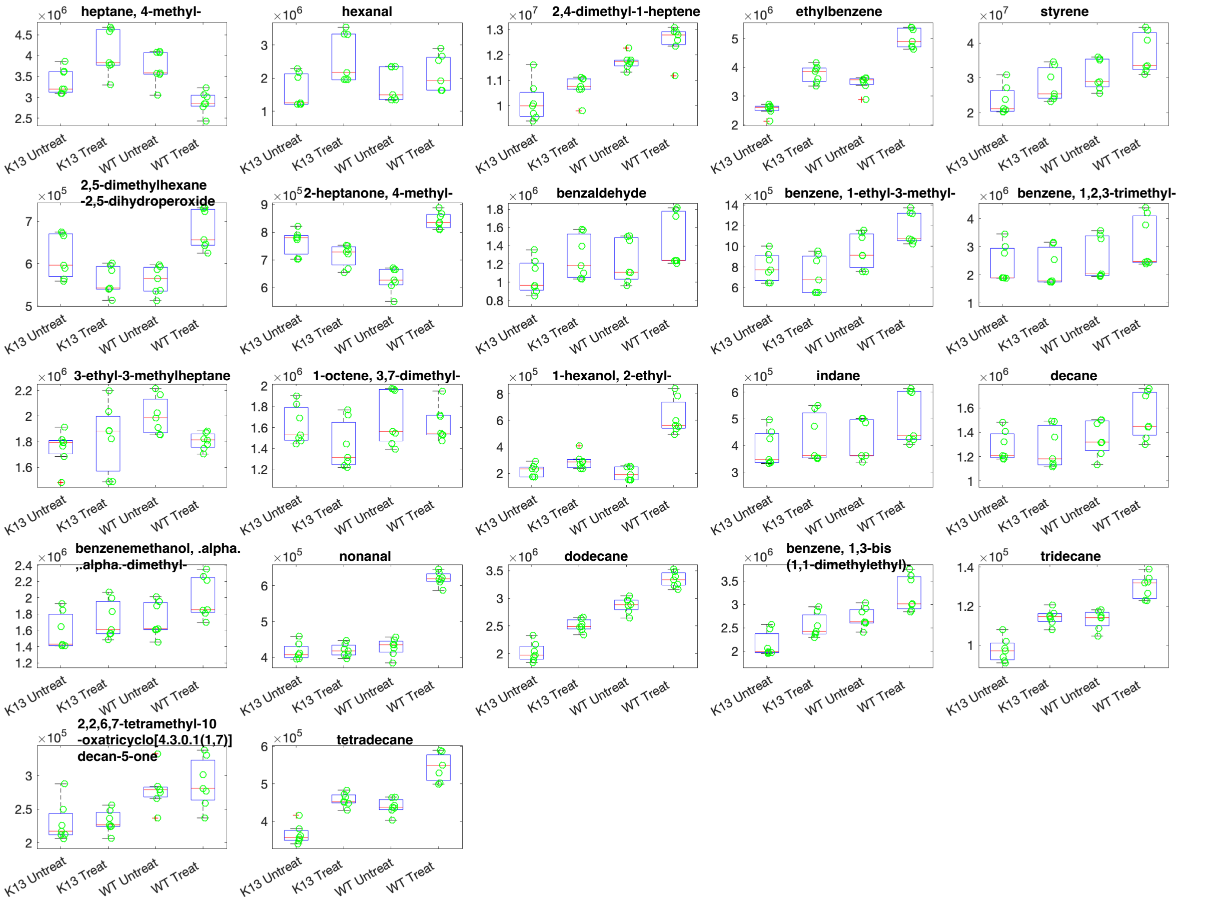

Figure S3: Distribution of the abundances of the compounds composing the Volatilome profile of artemisinin treated and untreated wild-type and resistant parasites.

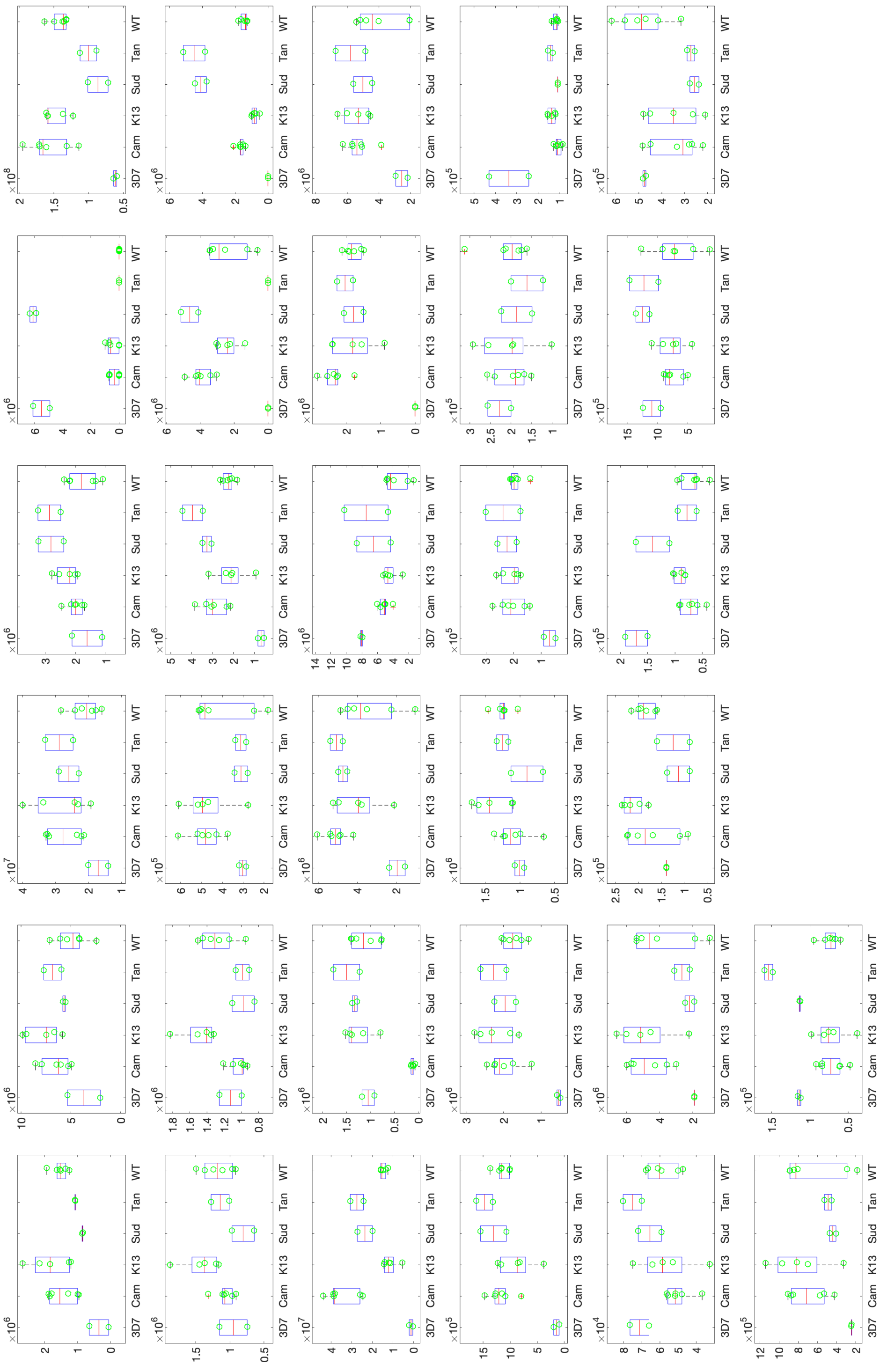

Figure S4 Distribution of the abundances of the compounds composing the Volatilome profile of lab-adapted and field isolated parasites cultured in Albumax.

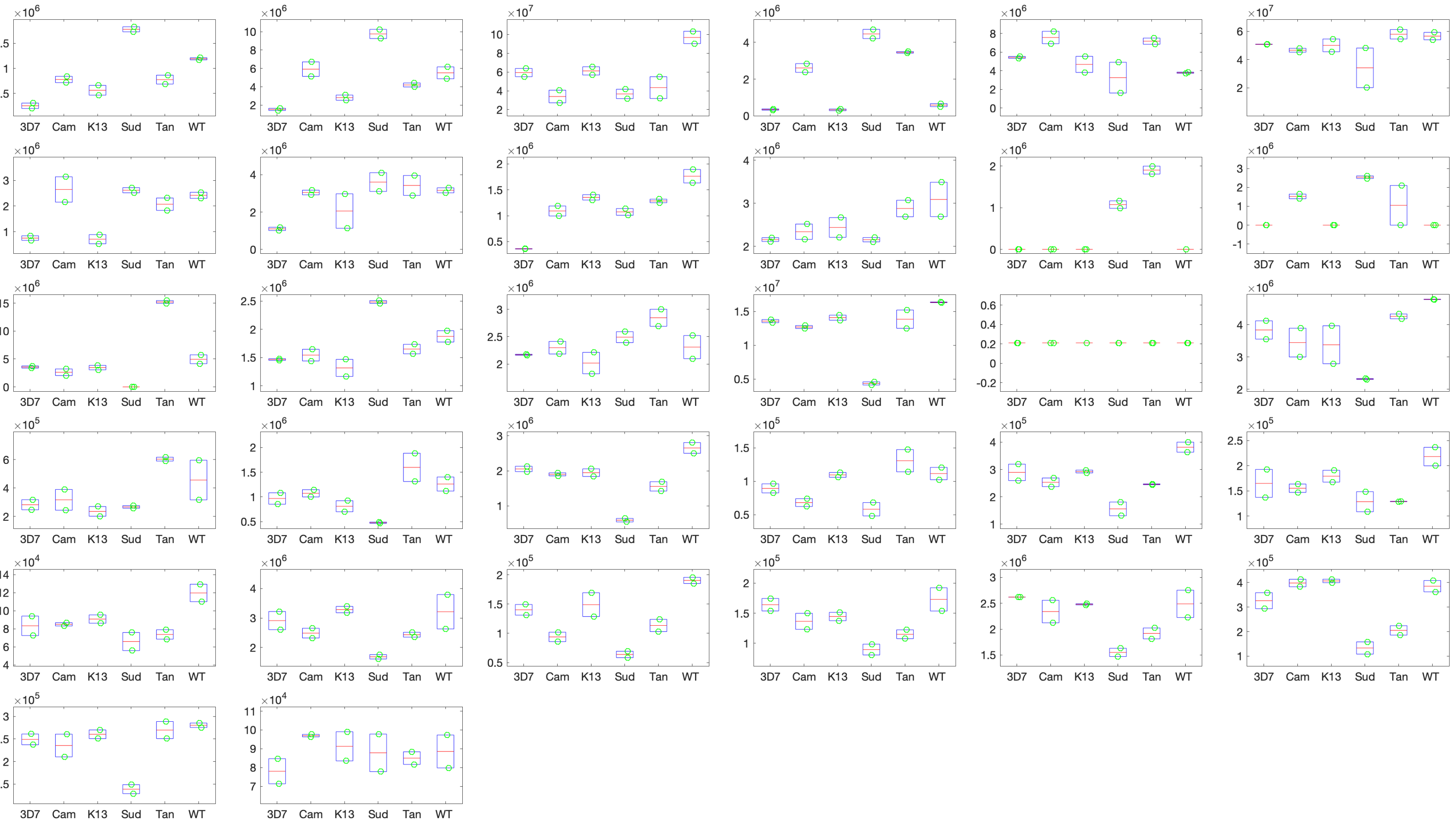
3D7
Cam
K13
Sud
Tan
WT

Figure S5: Distribution of the abundances of the compounds composing the Volatilome profile of lab-adapted and field isolated parasites cultured in Human Serum.
